# Biochar and Bioorganic Fertilizer Amendment Improved Soil Qualities and Altered Bacterial Communities in Quinoa Rhizosphere Soils of the Yellow River Delta

**DOI:** 10.3390/microorganisms14091878

**Published:** 2026-08-24

**Authors:** Meng Li, Yinyu Gu, Chuanjie Chen, Zongshuai Wang, Xiaohong Guo, Xiaoyan Liang, Kuihua Yi, Junlin Li, Dongyang Li, Haiyang Zhang

**Affiliations:** 1 Shandong Institute of Sericulture, Shandong Academy of Agricultural Sciences, Yantai 265503, China; 2 Crop Research Institute, Shandong Academy of Agricultural Sciences, Jinan 250100, China; 3 School of Resources and Environmental Engineering, Ludong University, Yantai 264025, China

**Keywords:** pyrogenic organic material, organic-based soil conditioner, microbial community, quinoa, saline-alkali soil

## Abstract

The application of biochar and bioorganic fertilizer (BOF) in agricultural systems has garnered increasing attention in recent years. Nevertheless, research remains scarce on the impacts of biochar and BOF on the rhizosphere microecological characteristics of saline-alkali soils. This research involved the execution and analysis of 16S rRNA sequencing using Illumina technology to explore how biochar, whether used alone or in conjunction with BOF, along with varying application rates, impacts the microbial community in the saline-alkali rhizosphere soil during quinoa cultivation. In the conducted field trial, sole BOF application, sole biochar application, and their combined application (referred to as BOFB) led to a substantial enhancement of 23.88%, 74.08–97.00%, and 188.88–220.59% in quinoa aerial biomass, respectively. Meanwhile, sole biochar application or biochar combined with BOF reduced soil electrical conductivity (EC) by 26.42–39.81%. Biochar and BOF significantly improved most soil parameters, with the exception of total phosphorus (TP). In comparison to the control (CK), the relative abundances of *Pseudomonas*, *Arthrobacter*, *Skermanella*, and *Bacillus* were elevated in the biochar and BOFB treatments, while *Sphingomonas* was more abundant in the BOF treatment. In addition, *Skermanella* exhibited a significant positive correlation with EC and available potassium (AK). Biochar exerted a stronger effect on soil bacterial community structure than BOF. Furthermore, the complexity of the bacterial community in biochar and BOFB treatments far exceeded that in the BOF and CK treatments. Overall, the application of biochar effectively reduced soil EC and improved soil fertility, enhanced bacterial community stability, and optimized bacterial community structure, thereby increasing quinoa aerial biomass. Under the conditions of this study, the optimal application rate for biochar was 15 t/ha, and the combined application of biochar and BOF produced superior effects relative to either amendment alone.

## 1. Introduction

Soil salinization poses a major global threat to agricultural sustainability and food security. Globally, over 1.38 billion hectares of land are affected by salinization, accounting for 10.7% of the total terrestrial area, and adversely impacting the livelihoods of over 2.4 billion people [1]. The Yellow River Delta (YRD) in China is a typical coastal saline-alkali region characterized by its unique saline-alkali soil properties. Formed via sediment deposition, it serves as an ecological buffer zone with unique and severe environmental constraints, including high soil salinity, inadequate drainage, and frequent secondary salinization. Nevertheless, effective exploitation can transform it into a valuable reserve of arable land [2]. The detrimental impacts of salinity on soil health are multifaceted; for instance, elevated salt concentrations induce osmotic stress that impedes plant water uptake, while excessive Na^+^ and Cl- exert cytotoxic effects on cellular metabolism and disrupt essential nutrient acquisition [3]. In addition, salinization disrupts the physical structure of the soil, hinders the accumulation of soil organic carbon, impairs the function of microbial communities, and interferes with the uptake of essential nutrients, thereby exerting a series of negative effects on crop yields [4,5]. Various amelioration strategies have been developed to tackle these challenges, which are broadly categorized into physical, chemical, and biological approaches [6]. Physical methods and chemical inorganic amendments typically entail significant economic costs and potential secondary environmental impacts [7]. Given these limitations, there is a growing recognition that sustainable utilization of saline-alkali land holds greater practical significance for achieving long-term agricultural resilience and food security, and the integrated application of improvement measures is moving toward the combined use of multiple measures [8,9]. Concurrent with the selection of appropriate crop species, the application of organic amendments, especially biochar and bioorganic fertilizers, has attracted considerable attention as a sustainable strategy for saline soil remediation [3,5].

Biochar, which is a carbon-rich material, is produced through the pyrolysis process of biomass sources such as agricultural waste, animal excrement, and timber in an oxygen-free environment [10]. With a wide range of excellent properties, such as porosity, large specific surface area, strong adsorption capacity, and good stability, biochar is increasingly recognized for its enormous potential in agricultural applications, including improving soil structure, sequestering carbon, and reducing greenhouse gas emissions [7]. In research on the remediation of saline-alkali soils, the addition of biochar can also reduce soil salinity and pH, increase total organic carbon content and water use efficiency, and improve soil physicochemical properties, consequently boosting crop yields [7,11]. Similarly, bioorganic fertilizers, produced by composting organic wastes with plant growth-promoting bacteria (PGPB), have demonstrated considerable potential in the rehabilitation of saline soil [8]. It has been confirmed that this approach can improve the structural characteristics and micro-ecological environment of saline soil, enhance the nutrient buffering capacity, and inhibit salt accumulation. Moreover, it combines the advantages of traditional organic fertilizers and microbial fertilizers, which can effectively improve fertilizer utilization efficiency and strengthen the remediation effect of saline-alkali soil [12].

The plant rhizosphere refers to a narrow soil zone surrounding plant roots, harboring diverse microbial communities and serving as a plant-shaped microenvironment and a highly active, complex interface between plants and soil [13]. The root system of plants is the main driving force for the aggregation of rhizosphere microbial communities. Plants attract microorganisms by releasing root exudates, which act as energy sources and chemical signals. Furthermore, factors including soil properties, plant genotypes, and environmental conditions also exert an influence on the assembly of rhizosphere microbial communities [3]. Meanwhile, microorganisms can conversely affect plant growth by synthesizing hormones and signaling molecules. This dynamic relationship between plants and rhizosphere microorganisms is jointly influenced by various biotic and abiotic factors. Furthermore, these interactions can be either beneficial or harmful, depending on the type of microorganisms involved. Consequently, the rhizosphere functions as the primary arena for nutrient exchange among plants, microorganisms, and soil, as well as a key site for microorganism-driven soil improvement [13].

Quinoa (*Chenopodium quinoa* Willd.), a facultative halophyte renowned for its high nutritional value and strong stress tolerance (including frost resistance, halotolerance, drought tolerance, and adaptability to nutrient-poor soils), has attracted increasing attention from researchers. Furthermore, quinoa represents a nutrient-rich and cost-effective food crop with great practical application potential. Consequently, quinoa was chosen as the test plant because it sustained stable growth across different treatment conditions and provided a solid basis for rhizosphere microbial investigations [14]. The combined application of biochar and bioorganic fertilizers in saline-alkali land, coupled with the cultivation of salt-tolerant plants, contributes to the restoration and improvement of saline-alkali land, thereby facilitating the sustainable utilization of saline-alkali soil. However, the reaction of the microbial community in the rhizosphere of quinoa to the use of biochar and bioorganic fertilizer in saline-alkali soil remains insufficiently explained. Therefore, this study was conducted on saline-alkali land to determine the effects of different application rates of biochar and bioorganic fertilizer on (i) soil nutrient status and physicochemical properties; and (ii) the rhizosphere bacterial community of quinoa in the target area. We hypothesize that utilizing suitable quantities of biochar and bioorganic fertilizer will enhance the chemical properties of soil and encourage the formation of soil bacterial communities, which in turn could help mitigate soil salinization.

## 2. Materials and Methods

### 2.1. Experimental Site and Sampling

The field experiment was conducted at the Dongying base of Shandong Academy of Agricultural Sciences (37.17° N, 118.36° E). This site is a typical saline-alkali land in the Yellow River Delta, and its specific soil texture and type are detailed in Gu et al. [15]. Prior to the sowing of quinoa in March 2020, biochar and bioorganic fertilizer were evenly spread on the soil’s surface, followed by deep raking of the soil to a depth of 20 cm to ensure uniform mixing of the amendments with the soil. The biochar utilized in the current experiment was provided by Taiyu Bioengineering Co., Ltd., Qixia, China. This material was produced from apple tree branches through slow pyrolysis under a constant temperature of 450 °C with a 24 h carbonization duration, and the basic chemical properties of biochar are shown in Table 1. The BOF adopted in this experiment was commercially obtained from Yangfeng Agricultural Technology Co., Ltd., Weifang, China. With mushroom residues and corn straw serving as the predominant feedstocks, this fertilizer was artificially fortified with Bacillus subtilis through microbial inoculation. Its core quality specifications are determined as follows: the effective viable bacterial count exceeded ≥5.0 × 10^9^ CFU g^−1^, the organic matter (OM) content was no lower than 60%, and the total nutrient concentration of nitrogen, phosphorus, and potassium (NPK) exceeded 6%.

The experimental protocol was based on a comparison between eight experimental treatments distributed in the field using a randomized design, with each plot having an area of 12 m × 8 m. The experimental treatments included the following: CK (control; neither biochar nor BOF), BOF (4.5 t/ha bioorganic fertilizer), B1 (7.5 t/ha biochar), B2 (15 t/ha biochar), B3 (22.5 t/ha biochar), BOFB1 (BOF + B1), BOFB2 (BOF + B2), and BOFB3 (BOF + B3). Quinoa seedlings were transplanted on April 16 at the three-leaf stage. To determine the plant height and fresh weight of the main stems, 9 uniformly developed quinoa plants were sampled from each treatment on 19 June. The fresh weight (FW) of the aerial biomass was measured immediately after measuring plant height (PH). Given the high germination sensitivity of quinoa seeds to water, persistent heavy rainfall before harvest resulted in a yield decrease.

### 2.2. Soil Sampling

Sample collection was carried out in May (M) and June (J), respectively. For each experimental plot, three quinoa rhizosphere soil samples were collected from the 0 to 20 cm soil layer. Excess soil was dislodged from root systems via vigorous shaking; only soil particles firmly bound to the roots were kept for study. The rhizosphere soils of three plants in each treatment were then pooled together and mixed thoroughly to form a single replicate for subsequent analysis. Samples were partitioned into two subsamples: one was snap-frozen in liquid nitrogen and dry-ice-shipped for high-throughput sequencing of microbial diversity; the other was air-dried, homogenized, and re-sieved through a 2 mm sieve to discard sand and root debris for soil chemical analyses.

### 2.3. Soil Chemical Properties

Soil pH and EC were measured at a soil-to-water ratio of 1:5 using a Magnetic Multi-parameter Water Quality Analyzer (DZS-708; INESA, Shanghai, Chinai) and a conductivity meter (DDS-307A; Shanghai Leici), respectively. Soil organic matter (SOM) contents were quantified using the potassium dichromate oxidation–external heating method with an SSM-5000A carbon analyzer (Shimadzu Corporation, Kyoto, Japan). Total phosphorus (TP) content was measured via the microwave digestion method and quantified using inductively coupled plasma optical emission spectrometry (ICP-OES, Agilent 720ES, Agilent Technologies, Santa Clara, CA, USA). Total nitrogen (TN) was determined by the dry-combustion method using an elemental analyzer (FLASH-2000, Thermo Scientific, Waltham, MA, USA). Available nitrogen (AN) was assessed by the alkaline-hydrolysis diffusion technique. Available phosphorus (AP) was extracted from soil samples using 0.5 M NaHCO_3_ and quantified by an ultraviolet spectrophotometer (TU-1910, Persee General, Beijing, China). Available potassium (AK) was extracted using 1 M ammonium acetate (pH 7.0) and determined via a flame photometer ( Model FP640, Shanghai Yuefeng, Shanghai, China).

### 2.4. Soil DNA Extraction

Genomic DNA was extracted using the E.Z.N.A.^®^ soil DNA Kit (Omega Bio-Tek, Norcross, GA, USA) and examined using a NanoDrop 2000 ultraviolet-visible spectrophotometer (Thermo Fisher Scientific, Waltham, MA, USA). The bacterial universal V3-V4 hypervariable region of the 16S rRNA gene was amplified using primers 338F (5′-ACTCCTACGGGAGGCAGCAG-3′) and 806R (5′-GGACTACHVGGGTATCTAAT-3′) [16]. After purification, the PCR products were quantified using a Quantus™ fluorometer (Promega Corporation, Madison, WI, USA). The purified amplicons were sequenced by Majorbio Bio-Pharm Technology Co., Ltd. (Shanghai, China) on the Illumina MiSeq PE300 platform (Illumina Inc., San Diego, CA, USA). Additional experimental details are shown in Table S1. All sequences have been deposited in the National Center for Biotechnology Information SRA database under accession number PRJNA1444555.

### 2.5. Data Analysis

Significant differences were determined by analysis of variance (ANOVA) and Duncan’s multiple-range test (*p* < 0.05) with SPSS Statistics software (version 26.0; IBM Corporation, New York, NY, USA). Specifically, α-diversity analysis was performed using Mothur software (v.1.30.2), and Venn and bar diagrams were plotted using R (ggvenn and ggplot2) (v.3.3.1); using the nst packages (3.1.10) in R packages (v.3.3.1), principal coordinates analysis (PCoA) was performed, and the normalized stochasticity ratio (NST) was calculated; Wilcoxon rank-sum tests for genus-level comparisons were corrected by the Benjamani–Hochberg FDR procedure, with adjusted *p* < 0.05 indicating statistical significance. Linear discriminant analysis effect size (LEfSe) analysis was implemented with an LDA score threshold of 2.0 to detect discriminant taxa via the Galaxy tool (http://galaxy.biobakery.org/ accessed on 12 February 2026). Niche analysis was carried out using spaa (version 0.2.2) in R (version 3.3.1). A neutral community model (NCM) was constructed using the Hmisc (version5.0.1), minpack.lm (version1.2.3), and getopt (version1.20.3) packages in R. Redundancy analysis (RDA) was performed using the RDA function in R (v.2.4.3). Network analysis was conducted using Networkx (version1.11); edges were filtered by Spearman correlation |r| ≥ 0.5 and *p* < 0.05. BugBase analysis was implemented through Bugbase (https://bugbase.cs.umn.edu/index.html, accessed on 1 February 2026). Data analyses were performed using the online Majorbio Cloud Platform (www.majorbio.com).

## 3. Results

### 3.1. Effects of Biochar and BOF Application on Quinoa Biomass and Soil Chemical Properties

**Figure 1:**
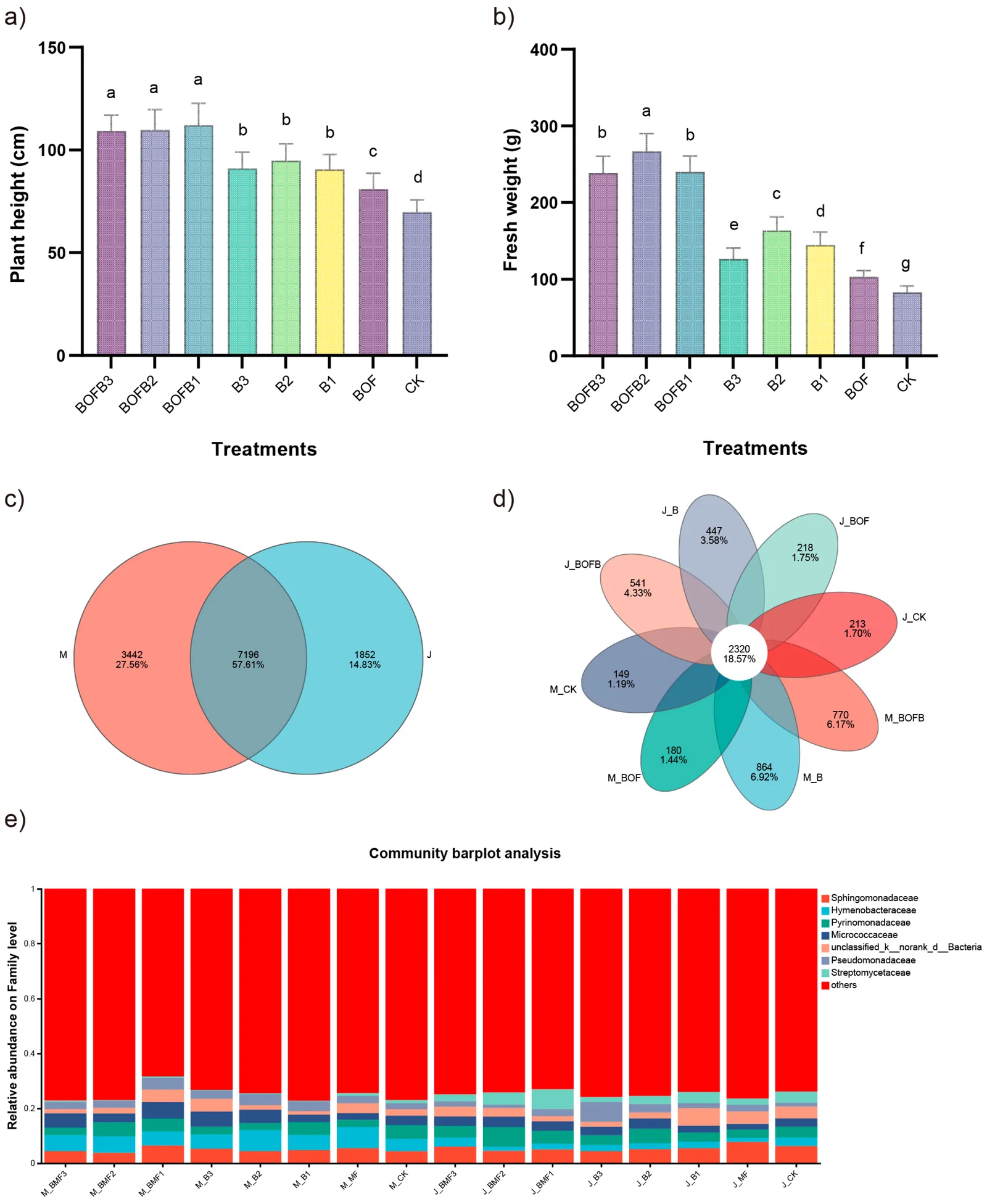
Quinoa agronomic traits and composition of bacteria under different treatments. (**a**) Plant height under different treatments. (**b**) Fresh weight under different treatments. (**c**) Venn diagram for the two-month samples. (**d**) Venn diagram for samples under different treatments. (**e**) Bar graphs representing relative abundance of major or dominant family across treatments. M, sampling in May; J, sampling in June. Different lowercase letters (a–g) above bars represent significant differences between treatments based on one‑way ANOVA with Tukey’s post‑hoc test at *P* < 0.05.

Compared with the control, the application of BOF alone increased quinoa plant height by 16.19%, whereas biochar alone increased it by 29.65–35.81%. The co-application treatments elevated plant height by 56.62–60.48%. In terms of aerial biomass, a similar trend to that for plant height was observed. BOF alone increased quinoa fresh weight by 23.88%, biochar alone increased it by 74.08–97.00% (with the highest value in B2), and co-application increased it by 188.78–220.59%, with the maximum increase recorded in BOFB2 (Figure 1a,b).

The responses of soil chemical properties to biochar and BOF application are summarized in Table 2 and Table 3. No significant difference in pH was observed between all experimental treatments, with a slight decrease observed in the BOFB. Whether in May (M) or June (J), the highest EC was observed in the CK, followed by the BOF, with no significant difference between these two treatments. However, both the sole application of biochar and its combined application with BOF significantly reduced EC compared with CK. For SOM, a significant increase was observed under all experimental treatments compared with CK, regardless of sampling time (May or June). As for TN, the order of TN concentration in May was as follows: high-concentration BOFB > low-concentration BOFB ≈ B > BOF > CK; in June, the TN concentration in high-concentration BOFB was significantly higher than that in the other treatments. In terms of TP, BOF showed the highest levels in May, while high-concentration B reached its peak in June. For AN, the order of AN concentration among all experimental treatments was BOFB > B ≈ BOF > CK, regardless of the sampling time (May or June). As for AP, the order of AP concentration among all experimental treatments was BOFB > B > BOF ≈ CK, regardless of whether it was in May or June. In terms of AK, the order of AK concentration among all experimental treatments in May was as follows: BOFB ≈ B > BOF ≈ CK; in June, the order of AK concentration was changed to B > BOFB > BOF > CK. With respect to the sampling month, there was no significant difference in pH and AN between May and June; EC significantly decreased by 11.36% in June compared with May, as did AP (11.03%) and AK (9.20%), while the concentrations of other soil nutrients (including SOM (12.46%), TN (13.83%), and TP (8.69%)) increased (Table S2).

### 3.2. Alpha-Diversity Index Analysis and Bacterial Composition

The gradual plateauing of rarefaction curves (Figure S2) indicated that the obtained sequencing data adequately covered the bacterial diversity across all samples. In May, significant differences were still observed between individual treatment plots; however, by June, neither richness nor diversity differed significantly among all treatments (Tables S3 and S4). A total of 2320 OTUs (18.57%) were shared across all treatments. The number of unique OTUs was higher in May than in June. Moreover, biochar-amended treatments harbored more unique OTUs than those without biochar addition. In May, the biochar application treatment harbored the highest number of unique OTUs, while the CK group presented the lowest value (Figure 1c,d).

The 12,490 OTUs were taxonomically assigned to 48 phyla, 150 classes, 404 orders, 705 families, 1591 genera, and 3509 species. The summed relative abundance of the four families (Sphingomonadaceae, Hymenobacteraceae, Pyrinomonadaceae, and Micrococcaceae) exceeded 13% of the total bacterial reads across all treatment groups (Figure 1e). Proteobacteria and Acidobacteriota dominated the bacterial community across all treatments, followed by Actinobacteriota and Bacteroidota. These four predominant phyla collectively accounted for over 70% of the total bacterial abundance in each treatment (Figure S3). The dominant bacterial genera for BOFB, B, BOF, and CK were *RB41*, *Pseudomonas*, *unclassified_k__norank_d__Bacteria*, and *RB41*, respectively. In addition, *RB41*, *norank_f__Vicinamibacteraceae*, *unclassified_k__norank_d__Bacteria*, *Pseudomonas*, *norank_f__Gemmatimonadaceae*, and *Pontibacter* prevailed as the predominant bacterial genera across all treatments and both growth stages (Figure S4).

### 3.3. Beta Diversity Analysis

**Figure 2:**
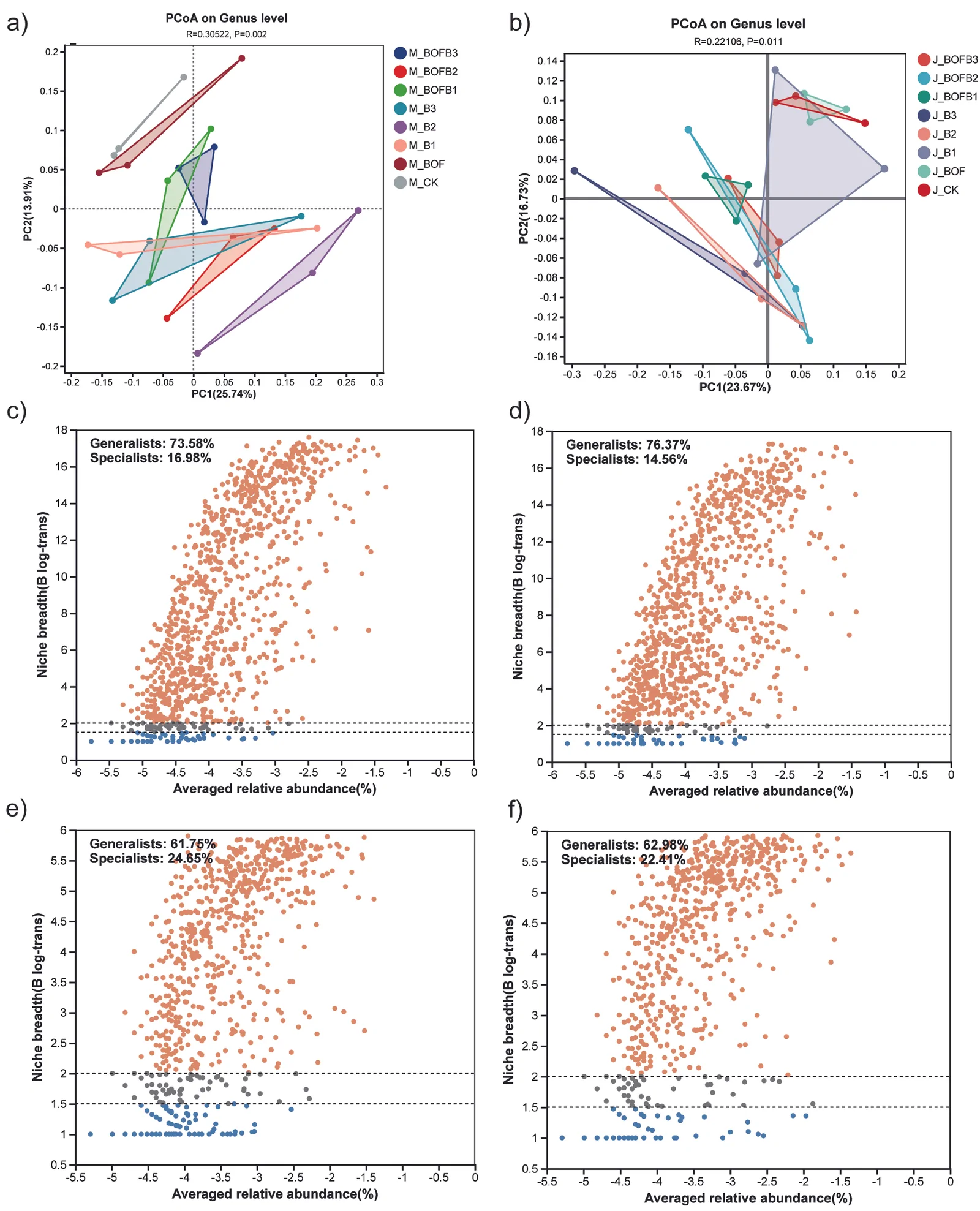
Beta diversity and community assembly of different treatments. (**a**) PCoA plot for samples under different treatments in May. (**b**) PCoA plot for samples under different treatments in June. (**c**) Scatter plot illustrating niche width for the BOFB group. (**d**) Scatter plot illustrating niche width for the B group. (**e**) Scatter plot illustrating niche width for the BOF group. (**f**) Scatter plot illustrating niche width for the CK group.

Based on the Bray–Curtis distance, PCoA was performed at the genus level to elucidate the major variations in bacterial community composition and abundance across samples. The PCoA ordination yielded two principal components explaining 25.74% (PC1) and 13.91% (PC2) of the variation among samples in May and 23.67% (PC1) and 16.73% (PC2) in June, respectively. Samples from each treatment clustered together, and regardless of whether it was May or June, the separation of BOFB and B relative to the CK was stronger than that of BOF relative to the CK. Additionally, B2 was the furthest from the CK, especially in May (Figure 2a,b).

### 3.4. Mechanisms of Bacterial Community Assembly

**Figure 3:**
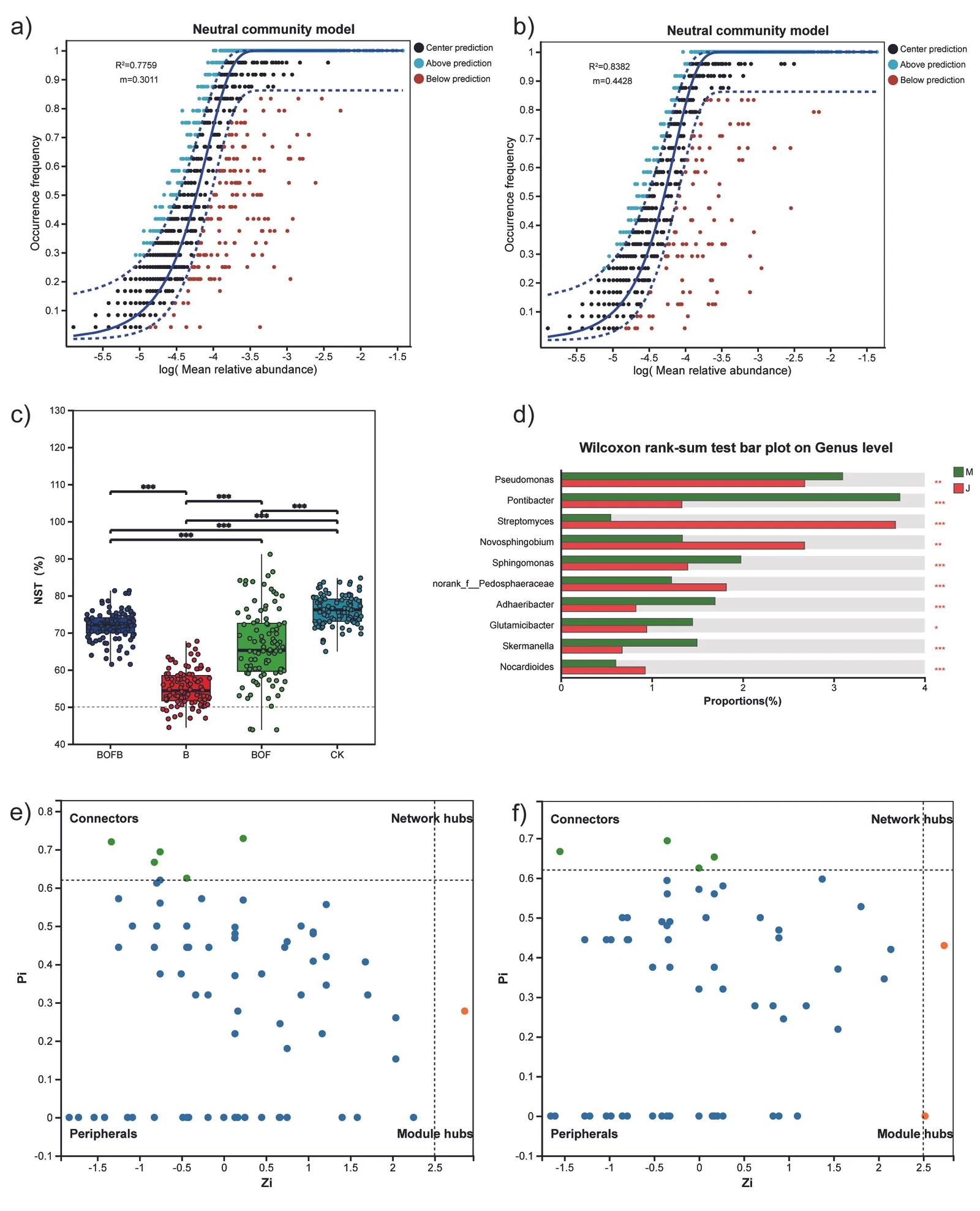
Bacterial assembly, community comparison, and screening of different treatments. (**a**) NCM for samples collected in May. (**b**) NCM for samples collected in June. (**c**) NST plot for samples under CK, B, BOF, and BOFB treatments. (**d**) Difference analysis between two months. (**e**) Zi-Pi plot for samples collected in May. (**f**) Zi-Pi plot for samples collected in June. * indicates *p* < 0.05, ** indicates *p* < 0.01, and *** indicates *p* < 0.001.

Ecological niche width is a critical factor shaping the relative importance of species sorting versus dispersal limitation. Taxa with broader niche widths typically possess higher metabolic plasticity. Broadly speaking, taxa characterized by high and low niche-width values are classified as generalists and specialists, respectively. Notably, the BOFB and B treatments harbored a higher proportion of generalist taxa (more than 73%) and a lower proportion of specialist taxa (less than 17%) relative to the BOF and CK treatments. The B treatment exhibited the highest proportion of generalists at 76.37% and the lowest proportion of specialists at 14.56%, while the BOF treatment showed the lowest proportion of generalists at 61.75% and the highest proportion of specialists at 24.65% (Figure 2c–f). Furthermore, the neutral community model (NCM) effectively fitted the relationships between the occurrence frequency of bacterial genera and their variations in relative abundance (Figure 3a,b). The R2 values for May and June were 0.7759 and 0.8382, respectively. The migration rate of the microbial taxa was lower in May (30.11%) than that in June (44.28%). These results indicated that the dispersal capacity of bacteria in June was higher than that in May. Furthermore, the normalized stochasticity ratio (NST) was calculated to quantify the contributions of deterministic and stochastic processes to microbial community assembly across different treatments (Figure 3c). NST values for bacterial communities in the BOFB, B, BOF, and CK treatments all exceeded the 50% threshold, with averages of 71.80%, 55.12%, 66.31%, and 76.10%, respectively. Most treatments exhibited distinctly higher NST values, providing strong evidence for prevailing stochasticity. Nevertheless, the B treatment yielded an NST of only 55.12%, which was just slightly above the 50% cutoff, representing a marginal case near the deterministic–stochastic boundary. Accordingly, stochastic processes dominated for most treatments, whereas the B treatment represented a marginal case near the deterministic–stochastic boundary.

### 3.5. Differential Bacteria Analysis

The relative abundances of core bacterial genera in May and June were compared (Figure 3d). The relative frequencies of *Pseudomonas*, *Pontibacter*, *Sphingomonas*, *Adhaeribacter*, *Glutamicibacter*, and *Skermanella* were higher in May than in June. Meanwhile, *Streptomyces*, *Novosphingobium*, *norank_f__Pedosphaeraceae*, and *Nocardioides* were significantly enriched in June. In addition, a Zi-Pi analysis was conducted to identify key species within microbial association networks (Figure 3e,f). *norank_f__Vicinamibacteraceae*, *norank_f__Saprospiraceae*, *Nitrospira*, *Massilia*, and *Gemmatimonas* were identified as connectors, while *Bryobacter* was a module hub in May; *norank_f__Microscillaceae*, *norank_f__Blastocatellaceae*, *norank_f__A4b*, and *Lactobacillus* were connectors, and *norank_f__Pedosphaeraceae* and *Arthrobacter* were module hubs in June. These results indicated that these genera were core microbiota.

LEfSe analysis was performed to screen for biomarker taxa with significant differences across treatments (Figure S5). In May, three bacterial taxa were significantly enriched in quinoa rhizosphere soil under CK and BOFB3 treatments. The B2 and B1 treatments followed, with two enriched taxa, respectively, and no differential bacterial taxa were identified in other groups. In June, the BOFB1 treatment yielded the highest quantity of enriched taxa (six) in quinoa rhizosphere soil, followed by B1 (four taxa) and BOF (two taxa). Notably, merely one enriched taxon was detected in the B3 and CK treatments.

### 3.6. Relationship Between Environmental Factors and the Microbial Community Data Matrix

Correlation network analysis was performed to explore the complexity of bacterial community interactions (Figure S6a–d), and its topological properties were computed (Table S5). Correlation network analysis based on the top 50 bacterial genera revealed that the BOFB and B treatments exhibited more complex ecological correlations than the BOF and CK treatments. The B treatment had the highest complexity and modular structure of correlation networks, with the average number of connections per node reaching 10.80. In contrast, the BOF treatment had the lowest, with an average of 4.47 connections per node. In addition, the B treatment had the maximum number of positive correlations, while the CK treatment showed the highest proportion of negative correlations.

**Figure 4:**
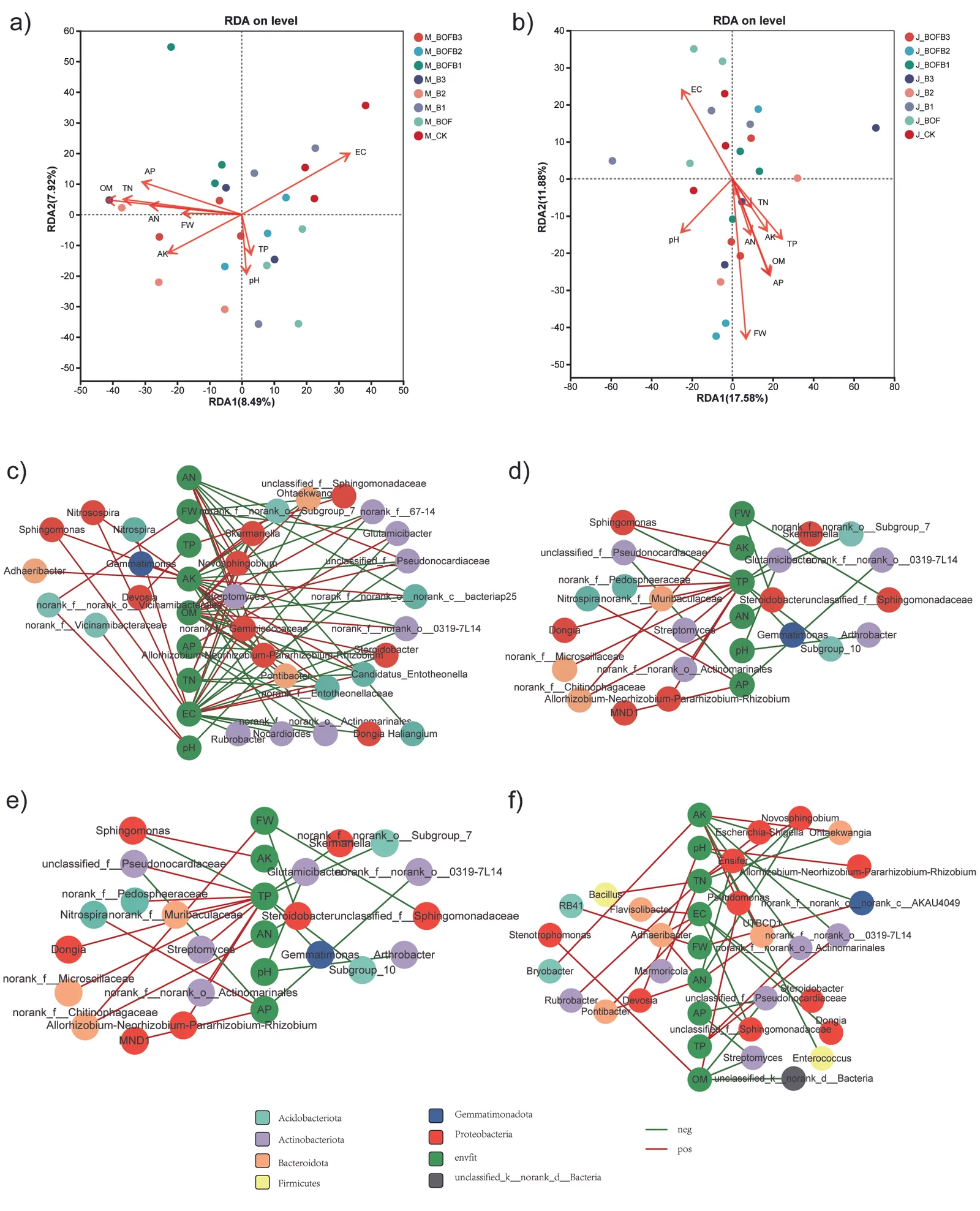
RDA and two-way Corr Network analysis of different treatments. (**a**) RDA plot for samples under different treatments in May. (**b**) RDA plot for samples under different treatments in June. (**c**) Two-way Corr Network plot for the BOFB treatment group. (**d**) Two-way Corr Network plot for the B treatment group. (**e**) Two-way Corr Network plot for the BOF treatment group. (**f**) Two-way Corr Network plot for the CK treatment group.

Redundancy analysis (RDA) revealed that variations in the bacterial community in May were significantly correlated with soil properties, including EC, SOM, AN, AP, and TN. By contrast, in June, bacterial community shifts were significantly correlated with FW (Figure 4a,b).

Furthermore, correlation network analysis was performed to explore the interactive relationships between bacterial communities and environmental factors (Figure 4c–f). In the BOFB treatment, EC and AK harbored the highest connections with bacteria (19), and *Skermanella*, *norank_f__Geminicoccaceae*, and *norank_f__Entotheonellaceae* had the highest connections with environmental parameters (5). With respect to the B treatment, TP possessed the highest connections with bacteria (13), and *Sphingomonas*, *Steroidobacter*, *norank_f__Chitinophagaceae*, and *unclassified_f__Pseudonocardiaceae* exhibited the highest connections with environmental parameters (2). Meanwhile, in the BOF treatment, the nodes with the highest connectivity between environmental parameters and bacteria were SOM (with six connections) and *unclassified_f__Sphingomonadaceae* (with five connections); in the CK treatment, the nodes with the highest connectivity were TN (nine connections) and *Novosphingobium*, *Pseudomonas*, and *UTBCD1* (each with three connections).

### 3.7. Potential Functional Consequences

The BugBase algorithm was applied to predict organism-level functional profiles and biologically interpretable phenotypic traits (Figure S7a,b). It should be noted that these phenotypic traits were inferred by BugBase based on 16S rRNA gene taxonomic information and pre-computed reference datasets rather than direct phenotypic or functional measurements of the microbial communities. In May, the functional phenotypes of Contains_Mobile_Elements and Forms_biofilms differed significantly across treatments. The B2 treatment had the highest relative abundance of Forms_biofilms, whereas CK showed the lowest relative abundance, with the order of relative abundance being B2 > B3 > BOFB2 > BOFB2 > B1 > BOFB3 > BOF > CK. However, only Facultatively_anaerobic showed significant differences among treatments in June.

## 4. Discussion

Quinoa has emerged as an important economic halophyte suitable for sustainable agriculture [2]. Currently, research on quinoa cultivation amended with organic materials remains limited. Woodchip biochar, either applied alone or combined with vermicompost, has been reported to promote quinoa growth under water stress conditions [16]. The plant growth and yield improved significantly via the application of acidified biochar in salt-affected soil [17]. Similarly, the plant height and aerial biomass significantly increased under biochar, alone and mixed with BOF in our study. Moreover, the overall effectiveness exhibited the following order: combined application > biochar alone > BOF alone > CK; this is consistent with findings from a related study on *Sesbania cannabina* [15]. Compared with biochar or BOF alone, BOFB provides a more direct and abundant nutrient supply to the soil. Moreover, the porous architecture of biochar can serve as a favorable shelter for soil microbial communities. When combined with the rich nutrients and beneficial bacteria in BOF, biochar synergistically promotes microbial survival and proliferation, collectively contributing to the superior performance of BOFB [15]. In the current study, 15 t/ha biochar yielded superior effects relative to the other two application rates. The discrepancies observed between our results and those of previous studies may be attributed to multiple factors, including biochar feedstock source, pyrolysis conditions, and initial soil fertility status [4].

Some studies have shown that biochar amendment generally increased soil pH [18,19]. In the present study, no significant differences were detected among treatments, irrespective of biochar or BOF amendment application. This may be attributed to the alkaline nature of biochar, for which its pH increases with rising pyrolysis temperature [20]. The biochar used in this study was pyrolyzed at 450 °C, thereby maintaining a relatively lower pH value. Biochar influences on soil EC are highly variable and rely on prevailing environmental and edaphic conditions [11]. In saline-alkali soil, amendment with 3% biochar decreased the EC of melon rhizosphere soil by 13.60% relative to the CK treatment [21]. There were also reports suggesting that biochar increased the soil EC by 8.8–44.8% [22] and reports indicating that EC was not significantly influenced by biochar addition [23]. There were even findings that 5% biochar treatment reduced soil EC, while the 10% biochar treatment increased it [24]. In this study, any treatment incorporating biochar significantly reduced EC; however, BOF alone had no effect on EC, which supported the findings of Wang et al. [25], differing from other reports due to factors such as biochar feedstock, carbonization parameters, and similar soil conditions [11,24]. In addition, as quinoa grew, the EC in June was significantly lower than that in May, suggesting that the combined effect of plants and biochar was more beneficial for saline-alkali soil remediation. Many studies have shown that soil nutrients (N, P, K, SOM, etc.) exhibited varying degrees of enhancement following the addition of biochar and BOF owing to their direct and indirect nutrient supply capabilities [26,27,28]. This field trial further verified these observations, indicating that the application of biochar and BOF significantly enhanced all indicators except TP. The combined application of biochar and other fertilizers outperformed the separate application of biochar alone or other fertilizers alone [2,29]. Our observations were in accordance with these results. Several factors may account for these observed results. First, the treatments differed in their inherent nutrient profiles. Second, the porous structure of biochar facilitated nutrient retention and controlled slow release over time. Third, BOF enhanced beneficial microbial communities, and the porous nature of biochar concurrently provided an optimal habitat for microbial activity [30].

Research indicated that biochar significantly increased the Ace and Chao indices of rhizosphere bacteria [31]. Our results similarly showed an increase in rhizosphere bacterial richness in May, but this increase dissipated by June. This finding contradicted the findings of You et al. [19], which indicated that biochar application showed no significant effect in the first year but improved the Chao index in the second year. Differences in effectiveness may stem from variations in biochar type, soil conditions, or application methods. As long as the treatment contained biochar, its unique OTUs exceeded those in both the BOF and CK groups and decreased over time, which indicated that biochar introduced more foreign bacteria into the rhizosphere soil, with only the fittest surviving. Proteobacteria and Acidobacteriota dominated the bacterial community at the phylum level across all treatments. Except for the treatment with high biochar content and BOF, which led to a lower relative abundance of Proteobacteria in June compared to the CK group, the relative abundance of Proteobacteria in all other treatments was higher than that in the CK group, which was consistent with the findings of Yan et al. [31]. This might contribute to the higher SOM and N content in all treatments compared to CK in this study, as Proteobacteria were key participants in carbon and nitrogen cycling processes [32,33]. In addition, the relative abundance of Acidobacteriota in June was higher in all treatments than in the CK group, which is consistent with the findings of You et al. [19]. This might also partly explain the higher nutrient content detected across all treatments, as Acidobacteriota acted as key contributors to nutrient cycling within soil microhabitats [34].

Amendment with biochar and bioorganic fertilizer reshaped the rhizosphere microbial community composition of quinoa. *RB41*, affiliated with the Acidobacteria, assists in maintaining the biogeochemical and metabolic foundation of soil [35], especially in C cycling [36]. Our study yielded similar results: As the most abundant bacterium, *RB41* was more abundant in the treatment combining biochar and bioorganic fertilizer than in the CK group in June. *Arthrobacter*, a well-known PGPB belonging to Actinobacteriota, has been reported to inhibit plant pathogens through the antagonistic effects of its secondary metabolites [37], increase proline content to cope with abiotic stress, and improve crop yields [38]. Consistently, *Arthrobacter* served as a modular hub in June, outperforming the CK group in terms of abundance in biochar-treated communities in both May and June and showing an inverse relationship with pH. However, inconsistently, it showed a positive correlation with biomass but an inverse relationship with SOM, and it had a lower abundance than the CK group in treatments with sole BOF application, which required further investigation. Among actinomycetes, *Streptomyces* represented the most valuable source of bioactive metabolites with promising biocontrol potential [39]. In addition, it has also been reported to have PGPB potential by stimulating the growth of host plants through phosphate solubilization, siderophore production, and nitrogen fixation [40]. Moreover, previous studies have reported that salt stress can be alleviated by triggering the ET-mediated defense pathway and activating the phenylpropanoid pathway [41]. Consistent with these findings, *Streptomyces* showed a significant negative correlation with EC and a positive correlation with AN. *Bryobacter*, a genus of Acidobacteriota, played key roles in promoting plant growth, inhibiting phytopathogens, decomposing soil organic matter, and regulating nutrient cycling [15]. In this study, it was a modular hub in May regardless of BOF application. The relative abundance of *Bryobacter* was higher in treatments with 15 t/ha of biochar than in the CK group, which was consistent with the results reported by Liu et al. [42], who noted that *Bryobacter* was elevated under wood and maize residue biochar treatments. Furthermore, *Bryobacter* was recognized as a pivotal microbial group regulating potassium accumulation in dryland soil [43], which supported our finding that this genus was positively correlated with soil AK. *Lactobacillus*, well known for its probiotic characteristics, has been reported to improve soil physicochemical properties, enhance soil enzyme activities, boost plant stress tolerance, reduce the bioavailability of soil heavy metals, and promote plant growth [44,45,46]. In the current study, *Lactobacillus* was detected in June. Regardless of BOF supplementation, biochar amendment significantly elevated the relative abundance of *Lactobacillus* in comparison with CK. *Skermanella*, a facultatively anaerobic-to-strictly aerobic Gram-negative genus, harbors nitrogenase-coding *nifDKH* but lacks the δ-subunit gene *anfG*, making it non-nitrogen-fixing [47], though it can grow in nitrogen-free media under microaerobic conditions [48]. In our study, *Skermanella* was uncorrelated with soil N content but positively correlated with EC and available potassium (AK), matching prior observations [49]. It was previously shown to dominate N-fixing bacterial communities and peak in biochar-amended calcareous purple soils [50]. Partially consistent with that pattern, *Skermanella* was enriched in BOFB3 in May but not different from CK in June; these inconsistencies may arise from differences in raw materials and experimental setups. The plant agent *Pseudomonas* has been extensively studied and is increasingly marketed as a plant PGPB and biological control agent [51]. Studies have demonstrated that this genus possesses beneficial potentials in nitrogen fixation, phosphorus solubilization, growth hormone secretion, and antimicrobial activity [52,53]. In the present study, the relative abundance of all treatments of *Pseudomonas* was higher than that in CK, especially in May, and was directly proportional to SOM and AK, indicating that biochar and BOF might be more conducive to the survival of *Pseudomonas*. *Sphingomonas* is well-known for its multifaceted functions, which range from biodegradation of environmental contaminants, abiotic stress tolerance, bioremediation, and the production of highly beneficial plant phytohormones [54] to playing a vital role in P cycling [55]. In partial agreement with earlier findings, BOF application led to a significant enrichment of *Sphingomonas* [56], and its relative abundance was directly proportional to AP. The above-mentioned genera have played a relatively important role in the interaction between biochar and bioorganic fertilizers with saline-alkali soil; however, further research is warranted to elucidate their specific functional mechanisms in the context of quinoa rhizosphere microecology and soil nutrient cycling.

In response to stress, plants can actively shape the assembly of rhizosphere microbial communities, thereby emphasizing their capacity to enhance growth through interactions with beneficial microbes [57]. A growing number of studies have categorized microorganisms into generalists and specialists based on their life-history strategies and ability to adapt to environmental conditions [58]. Traditionally, generalists with wide distribution and high population density exert greater influence on shaping ecosystem function and stability. In contrast, specialists occupied narrower ecological niches and maintained lower abundances, rendering them more sensitive to environmental disturbances [59]. In this study, compared with BOF and CK, BOFB and B had a higher proportion of generalist bacteria and a lower proportion of specialist bacteria, which indicated that increased nutrient levels and reduced salinity might provide a more favorable environment for species with strong adaptive capabilities. Stochastic processes played a more dominant role in microbial community assembly compared with deterministic processes [60]. The NST of the B treatment (55.12%) exceeded the threshold (50%); its value sits very close to the cutoff; other treatments showed considerably higher NST values, offering more robust evidence for stochastic processes governing community assembly. In the present study, stochastic processes dominated the assembly of bacterial communities for most treatments, which is consistent with the findings reported by Wang et al. [21]. Correlation network analysis demonstrated that biochar application alone harbored the highest complexity in the bacterial network, followed by BOFB treatment, and both were higher than those of BOF and CK, especially in terms of positive relationships. This result was consistent with the findings of Wang et al. [59], indicating that biochar supplementation could strengthen synergistic interactions and enhance the stability of the microbial network. Based on Bugbase functional prediction results, all treated groups exhibited significantly higher biofilm-forming capacity (Forms_biofilms) compared to the CK group in May, whereas this notable difference was no longer observed in June, which deserves further research. Although our predictions provide preliminary clues about community phenotypic shifts, direct metatranscriptomic or metagenomic sequencing would be required to validate the actual functional performances of rhizosphere microbiomes under biochar amendment.

Biochar substantially modifies rhizosphere bacterial community structure by altering soil physicochemical properties [7,11,15,16]. In line with earlier published findings, biochar amendment elevated soil nutrient levels, including soil organic carbon, N, P, and K. These available substrates can supply key resources for rhizosphere microorganisms. Meanwhile, biochar addition ameliorated saline-alkali soil conditions by reducing soil electrical conductivity, which further reshaped bacterial microhabitats. As a result, driven by enhanced soil nutrient availability, the co-application of biochar and bio-organic fertilizer yielded superior performance compared with single-amendment treatments of biochar or bio-organic fertilizer.

This field investigation was carried out for only one growing season at a single experimental site and employed one biochar type produced from a fixed feedstock under specific pyrolysis conditions. Severe heavy rainfall damaged reproductive plant tissues and led to the complete loss of reliable grain-yield data. Accordingly, the 15 t ha^−1^ biochar rate identified herein exhibits superior performance for above-ground biomass and plant height only under the present experimental settings and cannot be generalized as the optimal rate for grain yield improvement. Multi-location and multi-year field trials are therefore required to validate the agronomic potential of biochar amendment for saline-alkali soil crop production.

## 5. Conclusions

Under the saline-alkali field conditions of this study, biochar addition significantly lowered soil EC without altering soil pH. By contrast, sole bio-organic fertilizer application produced no significant changes in either soil EC or pH. Both amendments substantially increased rhizosphere soil concentrations of SOM, TN, AN, AP, and AK. Furthermore, biochar exerted a more pronounced influence on the composition and structure of soil bacterial communities compared with bio-organic fertilizer. With respect to bacterial community stability, biochar application also outperformed BOF application, and the latter outperformed CK. EC and SOM were crucial contributors to the regulation of bacterial distribution. Correlations between rhizosphere bacterial communities and soil chemical properties were strongest under the co-application of biochar and bio-organic fertilizer treatment, particularly for positive correlation relationships. Taken together, biochar application reduced soil EC and enhanced the stability of rhizosphere bacterial communities, with an optimal application rate of 15 t/ha under the present experimental settings. Both biochar and bio-organic fertilizer improved soil chemical properties, and their combined application outperformed their individual application. The findings of our study will provide a valuable reference for the remediation of salt-alkali soil using biochar and bio-organic fertilizer in the Yellow River Delta.

## Figures and Tables

**Table 1:** The basic chemical properties of biochar.

	pH	EC ms/cm	OC (%)	TN (%)	TP (%)	TK (%)
Biochar	7.49	0.36	20.8	0.83	0.21	0.67

**Table 2:** Soil chemical properties in May.

	pH	EC ms/cm	SOM (g/kg)	TN (g/kg)	TP (mg/kg)	AN (mg/kg)	AP (mg/kg)	AK (mg/kg)
M_BOFB3	8.09 ± 0.13a	0.468 ± 0.025 bc	21.7 ± 1.7 a	1.06 ± 0.12 a	718 ± 38 ab	60.7 ± 2.7 a	17.6 ± 0.8 a	330 ± 16 a
M_BOFB2	8.10 ± 0.14 a	0.491 ± 0.012 b	20.1 ± 0.1 ab	0.99 ± 0.14a b	635 ± 4 b	56.9 ± 8.0 ab	17.0 ± 2.3 ab	328 ± 5 a
M_BOFB1	8.19 ± 0.02 a	0.483 ± 0.018 b	20.5 ± 0.9 ab	0.97 ± 0.07 b	696 ± 48 ab	63.8 ± 0.4 a	17.7 ± 1.1 a	291 ± 14 cd
M_B3	8.39 ± 0.17 a	0.337 ± 0.001 d	17.4 ± 2.3 c	0.96 ± 0.12 b	655 ± 13 b	44.9 ± 2.0 c	16.4 ± 1.2 ab	318 ± 3 ab
M_B2	8.41 ± 0.28 a	0.311 ± 0.014 d	19.2 ± 2.2 b	0.97 ± 0.06 b	683 ± 22 ab	51.9 ± 1.9 bc	13.9 ± 0.5 c	278 ± 18 d
M_B1	8.34 ± 0.14 a	0.434 ± 0.054 c	15.2 ± 0.1 d	0.93 ± 0.01 b	684 ± 35 ab	49.8 ± 3.8 c	15.2 ± 0.1 bc	301 ± 5 bc
M_BOF	8.30 ± 0.08 a	0.500 ± 0.003 ab	13.7 ± 1.0 d	0.85 ± 0.09 c	759 ± 88 a	46.6 ± 3.4 c	11.0 ± 1.1 d	299 ± 7 e
M_CK	8.24 ± 0.42 a	0.536 ± 0.007 a	11.4 ± 0.2 e	0.75 ± 0.09 d	652 ± 26 b	35.1 ± 1.7 d	9.5 ± 0.3 d	229 ± 9 bc

Data represent the mean ± standard deviation (SD) of three biological replicates. Significant differences among treatments were evaluated using Duncan’s multiple-range test for mean separation at *p* < 0.05. Different lowercase letters within a row indicate significant differences (*p* < 0.05). M_ indicates samples collected in May; CK refers to the unamended control, B refers to biochar, BOF refers to bio-organic fertilizer treatment, and BOFB refers to combined biochar–BOF treatment. M_CK (control; neither biochar nor BOF, sampling in May), M_BOF (4.5 t/ha bioorganic fertilizer, sampling in May), M_B1 (7.5 t/ha biochar, sampling in May), M_B2 (15 t/ha biochar, sampling in May), M_B3 (22.5 t/ha biochar, sampling in May), M_BOFB1 (BOF + B1, sampling in May), M_BOFB2 (BOF + B2, sampling in May), and M_BOFB3 (BOF + B3, sampling in May).

**Table 3:** Soil chemical properties in June.

	pH	EC ms/cm	SOM (g/kg)	TN (g/kg)	TP (mg/kg)	AN (mg/kg)	AP (mg/kg)	AK (mg/kg)
J_BOFB3	8.15 ± 0.16 a	0.390 ± 0.013 b	26.8 ± 3.7 a	1.28 ± 0.04 ab	784 ± 89 abc	64.4 ± 0.9 a	14.8 ± 0.2 ab	274 ± 2 b
J_BOFB2	8.14 ± 0.40 a	0.349 ± 0.035 bc	29.7 ± 9.0 a	1.58 ± 0.55 a	674 ± 22 c	68.8 ± 14.5 a	16.5 ± 3.2 a	268 ± 1 b
J_BOFB1	8.30 ± 0.21 a	0.348 ± 0.024 bc	25.2 ± 0.1 ab	1.08 ± 0.08 bc	717 ± 14 bc	64.3 ± 3.1 a	13.7 ± 0.1 b	258 ± 4 b
J_B3	8.35 ± 0.18 a	0.319 ± 0.070 c	16.5 ± 1.8 cd	0.85 ± 0.04 c	861 ± 134 a	45.1 ± 0.9 b	13.6 ± 1.0 b	290 ± 1 a
J_B2	8.40 ± 0.12 a	0.333 ± 0.002 bc	18.9 ± 1.3 bc	0.98 ± 0.03 bc	835 ± 35 ab	51.3 ± 1.8 b	14.0 ± 0.4 b	295 ± 19 a
J_B1	8.41 ± 0.05 a	0.355 ± 0.026 bc	15.1 ± 1.1 cd	0.88 ± 0.04 c	708 ± 56 bc	48.5 ± 5.3 b	14.0 ± 0.4 b	290 ± 12 a
J_BOF	8.38 ± 0.09a	0.483 ± 0.007 a	13.3 ± 0.2 cd	0.95 ± 0.08 bc	686 ± 15 c	48.9 ± 3.0 b	9.8 ± 1.2 c	271 ± 2 b
J_CK	8.30 ± 0.37 a	0.530 ± 0.042 a	10.9 ± 0.1 d	0.94 ± 0.08 bc	692 ± 51 c	32.9 ± 1.4 c	8.7 ± 0.6 c	209 ± 3 c

Data represent mean ± standard deviation (SD) of three biological replicates. Significant differences among treatments were evaluated using Duncan’s multiple-range test for mean separation at *p* < 0.05. Different lowercase letters within a row indicate significant differences (*p* < 0.05). J_ indicates samples collected in June. J_CK (control; neither biochar nor BOF, sampling in June), J_BOF (4.5 t/ha bioorganic fertilizer, sampling in June), J_B1 (7.5 t/ha biochar, sampling in June), J_B2 (15 t/ha biochar, sampling in June), J_B3 (22.5 t/ha biochar, sampling in June), J_BOFB1 (BOF + B1, sampling in June), J_BOFB2 (BOF + B2, sampling in June), and J_BOFB3 (BOF + B3, sampling in June).

## Data Availability

The original contributions presented in the study are included in the article/Supplementary Material, further inquiries can be directed to the corresponding author.

## Supplementary Materials

The following supporting information can be downloaded at https://www.mdpi.com/article/10.3390/microorganisms14091878/s1. Table S1: PCR details; Table S2: Soil chemistry properties between months; Table S3: Diversity index of bacteria in May; Table S4: Diversity index of bacteria in June; Table S5: Correlation network analysis; Table S6: RDA; Figure S1: The experimental design; Figure S2: Rarefaction curves of Bacteria; Figure S3: Bar of phylum; Figure S4: Bar of genus; Figure S5: LEfSe analysis identifying discriminatory bacteria among samples under different treatments; Figure S6: Corr Network plot analysis; Figure S7: Bugbase functional phenotype prediction analysis.
