# Supplementary material for: Biochar and Bioorganic Fertilizer Amendment Improved Soil Qualities and Altered Bacterial Communities in Quinoa Rhizosphere Soils of the Yellow River Delta
Source: Microorganisms. 2026 Aug 24;14(9):1878. doi: 10.3390/microorganisms14091878 (PMC13609551; doi:10.3390/microorganisms14091878)
Supplement: Supplementary file 1 [file microorganisms-14-01878-s001.zip › microorganisms-4474059-supplementary matierial.pdf]

Table S1 PCR details

	Experiment details
PCR mixtures	The PCR mixtures contained 4 μL of 5x TransStartFastPfu buffer, 2 μL of 2.5 mM dNTPs, 0.8 μL of each primer (5 μM each), 0.4 μL of *TransStart*FastPfu DNA Polymerase, 10 ng template DNA, adding ddH_2O to a final volume of 20 μL. All reactions were performed in triplicate.
PCR cycling conditions	PCR cycling conditions included an initial denaturation at 95°C for 3 min, 27 cycles of denaturing at 95°C for 30 s, annealing at 55°C for 30 s, and extension at 72°C for 45 s, followed by a single extension at 72°C for 10 min and a continued hold at 4°C.
Splicing and quality control	Raw sequence reads were demultiplexed, quality-filtered by fastp version 0.20.0, and merged by FLASH version 1.2.7. Operational taxonomic units (OTUs), with a 97% similarity cut-off, were clustered using UPARSE v.7.1, and chimeric sequences were identified and removed. The taxonomy of each OTU representative sequence was analyzed by RDP Classifier v.2.2 against the 16S rRNA database using a confidence threshold of 0.7.

Table S2 Soil chemistry properties between months

	pH	EC ms/cm	SOM (g/kg)	TN (g/kg)	TP (mg/kg)	AN (mg/kg)	AP (mg/kg)	AK (mg/kg)
May	8.26±0.21a	0.44±0.08a	17.41±3.68b	0.94±0.12b	685.23±51.86b	51.22±9.38a	14.78±3.1a	296.73±32.53a
June	8.3±0.22a	0.39±0.08b	19.58±7.21a	1.07±0.29a	744.77±87.82a	53.02±12.45a	13.15±2.69b	269.42±27.12b

Table S3 Diversity index of bacteria in May

	Sobs	Ace	Chao	Shannon	Simpson	Coverage
M_BOFB3	3066±254abc	4014±365abc	3968±370ab	6.67±0.17a	0.0041±0.0012bc	0.97±0.003ab
M_BOFB2	3376±98a	4412±152a	4324±123a	6.79±0.07a	0.0039±0.0006c	0.967±0.001b
M_BOFB1	2815±285c	3730±435c	3679±445b	6.34±0.09b	0.0075±0.0011a	0.972±0.004ab
M_B3	3175±21abc	4211±57abc	4113±106ab	6.55±0.11ab	0.0067±0.0021ab	0.968±0.001ab
M_B2	3282±111ab	4330±225ab	4263±211a	6.65±0.06a	0.0051±0.0014abc	0.967±0.002ab
M_B1	2991±188abc	3891±257abc	3847±292ab	6.59±0.1ab	0.0048±0.0008abc	0.971±0.002ab
M_BOF	2961±339bc	3841±416bc	3800±391ab	6.64±0.29a	0.0041±0.0016bc	0.972±0.003a
M_CK	2880±100bc	3854±122bc	3826±131ab	6.53±0.14ab	0.0056±0.0015abc	0.971±0.001ab

Table S4 Diversity index of bacteria in June

	Sobs	Ace	Chao	Shannon	Simpson	Coverage
J_BOFB3	2893±67a	3843±78a	3837±36a	6.58±0.07a	0.0045±0.0007a	0.971±0.001ab
J_BOFB2	2799±208a	3670±265a	3613±275a	6.4±0.2a	0.0072±0.0015a	0.973±0.002ab
J_BOFB1	2846±123a	3789±133a	3794±186a	6.43±0.17a	0.0067±0.0024a	0.972±0.001ab
J_B3	2880±284a	3765±368a	3693±347a	6.49±0.48a	0.0072±0.0068a	0.973±0.002ab
J_B2	2872±193a	3810±254a	3751±231a	6.54±0.29a	0.0055±0.0026a	0.972±0.002ab
J_B1	2788±191a	3620±212a	3551±194a	6.43±0.25a	0.0078±0.0048a	0.974±0.002a
J_BOF	2932±97a	3820±134a	3786±115a	6.59±0.05a	0.0053±0.0015a	0.972±0.001ab
J_CK	2973±29a	3939±52a	3908±86a	6.55±0.05a	0.0053±0.0006a	0.971±0.001b

Table S5 Correlation network analysis

	Node numbers	Edge numbers	Node average degree	Positive edges	Negative edges
BOFB	49	227	9.27	136	91
B	50	270	10.8	200	70
BOF	47	105	4.47	62	43
CK	48	127	5.29	69	58

Table S6 RDA analysis

	May		June	
	r2	P_value	r2	P_value
pH	0.09978	0.351	0.13038	0.222
EC	0.45153	0.001	0.22047	0.084
OM	0.57981	0.001	0.18659	0.119
TN	0.4446	0.003	0.02423	0.742
TP	0.04671	0.592	0.15173	0.209
AN	0.25997	0.037	0.05618	0.538
AP	0.34353	0.013	0.19502	0.115
AK	0.20047	0.09	0.08682	0.373
FW	0.10356	0.314	0.37353	0.005

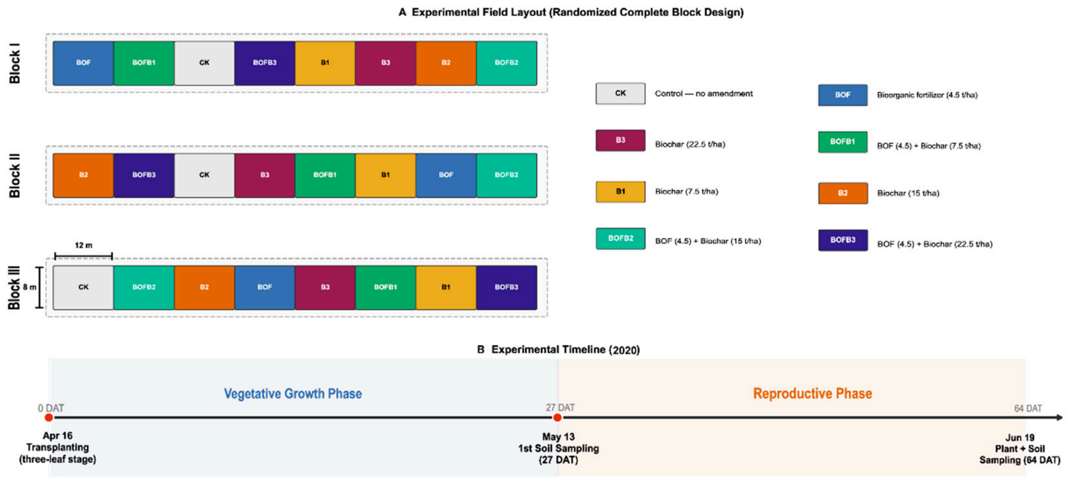

Figure S1 The experimental design

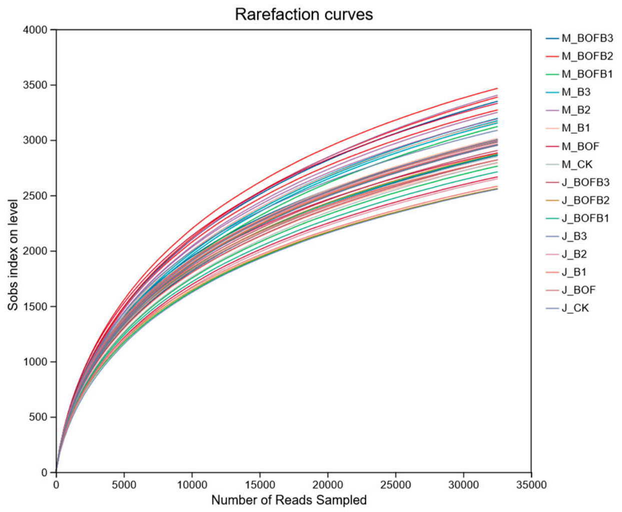

Figure S2 Rarefaction curves of Bacteria

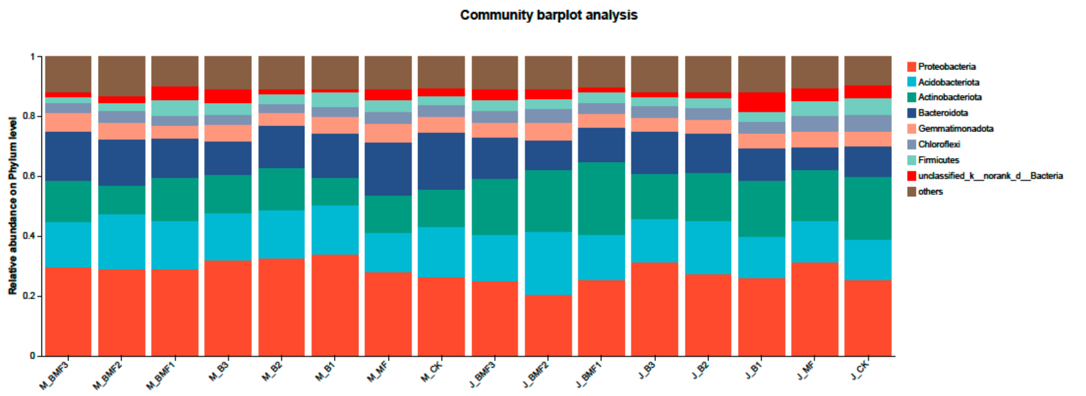

Figure S3 Bar of phylum

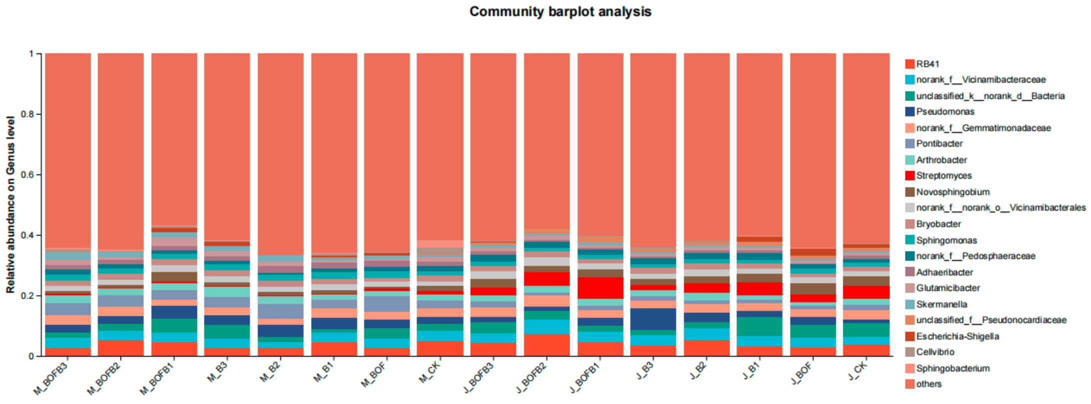

Figure S4 Bar of genus

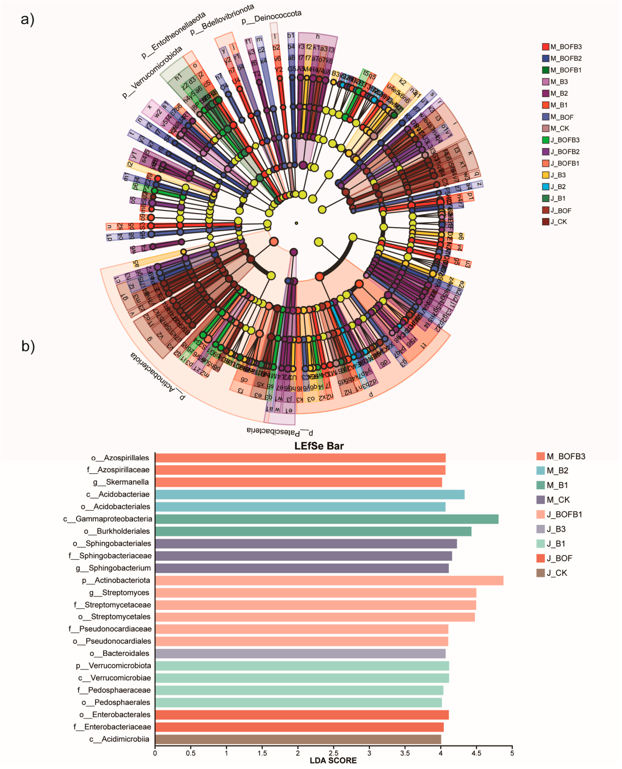

Figure S5 LEfSe analysis identifying discriminatory bacteria among samples under different treatments.

(a) LEfSe cladogram showing taxonomic biomarkers. (b) Bar plot of LDA effect sizes for

differentially enriched taxa.

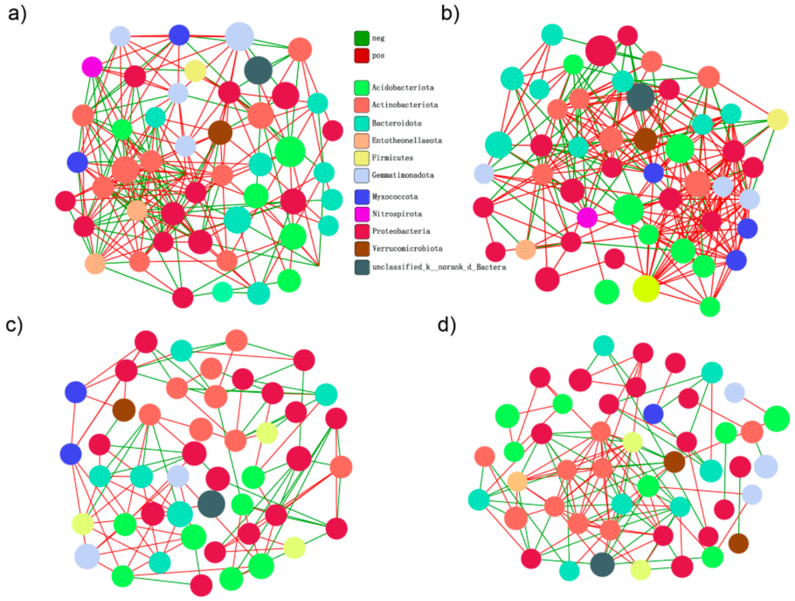

Figure S6 CorrNetwork network plot analysis.

(a) CorrNetwork network plot for the BOFB treatment group. (b) CorrNetwork network plot for the B treatment group. (c) CorrNetwork network plot for the BOF treatment group. (d) CorrNetwork network plot for the CK treatment group.

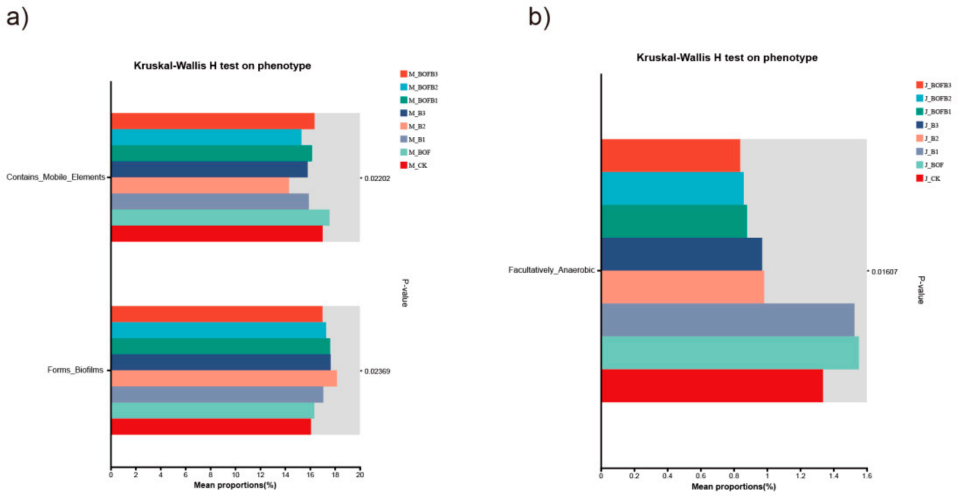

Figure S7 Bugbase functional phenotype prediction analysis.

(a) Bugbase functional phenotype prediction for samples collected in May. (b) Bugbase functional phenotype prediction for samples collected in June.
